# Association of post-treatment hypoalbuminemia and survival in Chinese patients with metastatic renal cell carcinoma

**DOI:** 10.1186/s40880-017-0214-7

**Published:** 2017-05-18

**Authors:** Wen Cai, Jin Zhang, Yonghui Chen, Wen Kong, Yiran Huang, Jiwei Huang, Lixin Zhou

**Affiliations:** Department of Urology, Renji Hospital, School of Medicine, Shanghai Jiao Tong University, 160 Pujian Rd., Pudong District, Shanghai, 200127 P. R. China

**Keywords:** Metastatic renal cell carcinoma, Post-treatment hypoalbuminemia, Prognosis, Tyrosine kinase inhibitors

## Abstract

**Background:**

Hypoalbuminemia adversely affects the clinical outcomes of various cancers. The purpose of this study was to estimate the prognostic value of hypoalbuminemia 3–5 weeks after treatment in patients with metastatic renal cell carcinoma (mRCC) who received sorafenib or sunitinib as first-line treatment.

**Methods:**

In this single-center, retrospective study, we assessed the progression-free survival (PFS) and overall survival (OS) of 184 mRCC patients who received first-line sorafenib or sunitinib treatment. PFS and OS were compared between patients with post-treatment hypoalbuminemia (post-treatment albumin level <36.4 g/L) and those with normal post-treatment albumin level (albumin level ≥36.4 g/L). The Memorial Sloan Kettering Cancer Center (MSKCC) risk model stratified mRCC patients into three risk categories. Prognostic values of all patient characteristics including MSKCC risk category were determined by using univariate and multivariate Cox regression models. Prognostic value was further determined using the Harrell concordance index and receiver operating characteristic curve analysis.

**Results:**

The median PFS and OS of the 184 patients were 11 months (95% confidence interval [CI] 9–12 months) and 23 months (95% CI 19–33 months), respectively. Patients with post-treatment hypoalbuminemia had significantly shorter median PFS (6 months [95% CI 5–7 months]) and OS (11 months [95% CI 9–15 months]) than patients who had normal post-treatment albumin levels (PFS: 12 months [95% CI 11–16 months], *P* < 0.001; OS: 31 months [95% CI 24–42 months], *P* < 0.001), respectively. Multivariate analysis showed that post-treatment hypoalbuminemia was an independent predictor of PFS (hazard ratio [HR], 2.113; 95% CI 1.390–3.212; *P* < 0.001) and OS (HR, 2.388; 95% CI 1.591–3.585; *P* < 0.001). Post-treatment hypoalbuminemia could also be combined with the MSKCC risk category for better prediction about OS. The model that included post-treatment hypoalbuminemia and MSKCC risk category improved the predictive accuracy for PFS and OS (c-index: 0.68 and 0.73, respectively) compared with the basic MSKCC risk model (c-index: 0.67 and 0.70, respectively). The prognostic values for PFS and OS of the integrated MSKCC risk model involving post-treatment hypoalbuminemia were significantly more accurate than the basic MSKCC risk model using likelihood ratio analysis (both *P* < 0.001).

**Conclusions:**

Post-treatment hypoalbuminemia can be considered an independent prognostic factor for patients with mRCC who undergo first-line treatment with tyrosine kinase inhibitors. Additionally, integrating post-treatment serum albumin level into the basic MSKCC risk model can improve the accuracy of this model in predicting patient overall survival and progression-free survival.

## Background

Approximately 25%–30% of patients with renal cell carcinoma (RCC) are found to have metastatic lesions at their first diagnosis [1]; the remaining patients eventually develop metastatic RCC (mRCC) [2]. RCC is particularly insensitive to chemotherapy or radiotherapy, and only 20% of RCC showed sensitivity towards the standard cytokine regimen; the disease control is limited, and the median overall survival (OS) of RCC patients was less than approximately 12 months [3]. In China, tyrosine kinase inhibitors (TKIs), such as sorafenib and sunitinib, are widely used in routine clinical settings as first- and second-line therapies, respectively, for patients with mRCC [4–9]. The Memorial Sloan Kettering Cancer Center (MSKCC) risk model focuses primarily on five principal adverse prognostic factors: Karnofsky performance status (KPS) score less than 80; serum lactate dehydrogenase (LDH) concentration more than 1.5 times the upper limit of normal; low hemoglobin (<130 g/L in men, <115 g/L in women); serum calcium concentration over 0.1 g/L; and the time from diagnosis to treatment less than 1 year [10]. The MSKCC risk model stratifies mRCC patients based on the number of adverse prognostic factors into three risk categories: favorable, intermediate, and poor risk groups; the poor risk group had poor disease outcomes after interferon treatment [11]. Furthermore, pre-treatment neutrophil-to-lymphocyte ratio (NLR) and C-reactive protein level were included as effective prognostic factors in the MSKCC risk model [12–15]. In addition, absolute neutrophil count, platelet count, leukocyte count, and serum hemoglobin, LDH, phosphatase alkaline, and calcium levels were shown to be independent risk factors [16, 17]. Therefore, investigating new prognostic factors related to the survival outcome of mRCC patients who receive targeted therapy is of paramount importance.

In urological and other major malignancies, nutritional malfunction (usually presented as low body mass index [BMI] and decreased weight and serum albumin level) is related to high occurrence rates of postoperative complications and disease-related death or short OS [18–24]. Nutritional malfunction suppresses albumin synthesis, causing hypoalbuminemia, which may have an adverse effect on the overall effectiveness of TKIs in mRCC patients and may result in poor survival outcomes. Studies have shown that serum albumin is an important predictor for the outcome of patients with mRCC [25–28]. Since the half-life of serum albumin is approximately 20 days, we focused on the serum albumin level at 3–5 weeks after onset of targeted therapy, which would indicate the change in albumin levels after treatment. In addition, in patients with mRCC who receive first-line TKIs, the effect of post-treatment serum albumin level on the outcomes of mRCC patients remains elusive. Thus, in this study, we investigated the prognostic implications of post-treatment serum albumin levels to establish a novel risk stratification model for patients with mRCC who receive sorafenib or sunitinib as first-line treatment.

## Patients and methods

### Study population

In this retrospective, single-center study, we analyzed the electronic medical records and laboratory results of all patients treated between March 2006 and September 2015 in the Department of Urology at Renji Hospital, affiliated to Shanghai Jiao Tong University School of Medicine, Shanghai, China. The protocol conformed to the principles of the Declaration of Helsinki and was approved by the institutional ethics committee of Renji Hospital. This study included mRCC patients (1) who received sorafenib or sunitinib as first-line therapy and who had a KPS score of 70–100 and (2) who had the records of serum albumin levels before and after treatment. The patients were excluded from the study if they did not meet the above criteria or had unstable or severe cardiac disease, uncontrolled brain metastases, concurrent malignancies, or incomplete data files.

### Treatment

Sorafenib or sunitinib was used as first-line treatment for all mRCC patients. Sorafenib were administered at a dose of 300 and 400 mg orally twice daily for patients with low and high albumin levels, respectively, in a 4-week cycle continuously until disease progression, intolerable adverse events, or death. Similarly, sunitinib were administered at a dose of 37.5 and 50 mg orally once daily for patients with low and high albumin levels, respectively, in a 6-week cycle (4-week on, 2-week off—a 4/2 schedule) until disease progression, intolerable adverse events, or patient withdrawal.

National Cancer Institute Common Terminology Criteria for Adverse Events version 3.0 (NCI-CTC 3.0) were used for diagnosis [29] and grading of treatment-related adverse events, based on which the drug dose were modified. Patients were restricted to be treated at the onset of disease progression or unacceptable toxicity (that is, the toxicity needs suspending targeted therapy of at least 4 weeks for recovery to a permissible level despite two dose reductions), as determined by the Response Evaluation Criteria in Solid Tumors (RECIST) [30].

### Clinicopathologic evaluation and laboratory assays

Information on patient demographic characteristics was retrieved from the medical record database. One week before treatment, a detailed examination, including medical history taking and physical examination as well as complete blood count, NLR, routine organ function tests, computed tomography and magnetic resonance imaging scans, and histological differentiation graded according to the Fuhrman nuclear grading system, was performed. Serum albumin levels were measured in 1 week before and 3–5 weeks after the onset of targeted therapy. The laboratory parameters for this study and information on occurrence and severity of adverse events were obtained from patient medical records.

### Safety and response rate assessments

Primary endpoints for this study were PFS and OS. PFS was defined as the duration from the onset of targeted therapy to disease progression or death as assessed by the treating physician or the last visiting day recorded if the disease did not progress. OS was defined as the duration from the onset of targeted therapy to death or the last visiting day recorded if the patient was alive. The associations of hypoalbuminemia with clinicopathologic characteristics and prognosis of patient with mRCC were determined by using the Cox proportional hazards model. Tumor response was evaluated using RECIST. NCI-CTC 3.0 was used for classification of severity of adverse events.

### Follow-up

All patients were followed up and assessed by outpatient service to estimate the tolerance and adverse effects within 1–2 weeks of the onset of targeted therapy, and then their disease statuses were assessed every month or any time they felt discomfort after the treatment.

### Statistical analysis

SAS version 9.1 (SAS Corporation, Cary, NC, USA) and SPSS version 18.0 (SPSS Inc., Chicago, IL, USA) were used for statistical analyses. Pre-treatment and post-treatment serum albumin levels were compared by using *t* test. Continuous variables are presented as median (interquartile range); categorical variables are presented as number of patients followed by percentages and were analyzed by using Pearson’s Chi square test. Time-dependent receiver operating characteristic (ROC) analysis was performed to determine the best cutoff point of serum albumin level before and after treatment. In the present study, the post-treatment serum albumin level lower than the cutoffs determined by ROC analysis was defined as post-treatment hypoalbuminemia. The Kaplan–Meier method was used to estimate the survival, and the log-rank test was used to compare the PFS and OS between high and low serum albumin groups. The Cox proportional hazards model was used to estimate the prognostic value of clinical variables, including age, sex, history of cytokine and surgical treatment, pathologic type, number of metastatic sites, MSKCC risk category, Fuhrman grade, NLR, and pre-treatment and post-treatment serum albumin levels. All statistical tests were two-sided, and *P* values less than 0.05 were considered statistically significant. Predictive analysis was conducted using the Harrell concordance index (c-index) to calculate predictive ability. The c-index was built based on a training set with the R package “survival.” Finally, time-dependent ROC analysis was conducted after adding the post-treatment hypoalbuminemia to the basic MSKCC risk model.

## Results

### Patient demographics and clinicopathologic characteristics

We reviewed the medical records of 266 consecutive patients with mRCC who received TKIs. After excluding patients with incomplete data, 184 patients (137 men [74.5%] and 47 women [25.5%]), with a median age of 60 years (range 24–82 years), were included in the cohort. Of these, 38 patients constituted the post-treatment hypoalbuminemia (<36.4 g/L) group, and 146 patients constituted the normal post-treatment albumin level (≥36.4 g/L) group. Most patients were sorted to Furman grade 1–2 (56.5%) and favorable MSKCC risk category (45.1%). Sorafenib and sunitinib were administered as first-line therapy to 112 (60.9%) and 72 (39.1%) patients, respectively. Table 1 shows the distribution of baseline demographics in the two groups.

**Table 1:** The baseline and clinicopathologic characteristics of 184 patients with metastatic renal cell carcinoma

Variable	No. of patients (%)	Post-treatment hypoalbuminemia group (<36.4 g/L)	Normal post-treatment albumin level group (≥36.4 g/L)	*P* value
Sex				0.589
Men	137 (74.5)	27 (71.1)	110 (75.3)	
Women	47 (25.5)	11 (28.9)	36 (24.7)	
Age (years)				0.215
<65	141 (76.6)	32 (84.2)	109 (74.7)	
≥65	43 (23.4)	6 (15.8)	37 (25.3)	
Pathologic type				0.060
ccRCC	179 (97.3)	35 (92.1)	144 (98.6)	
nccRCC	5 (2.7)	3 (7.9)	2 (1.4)	
History of nephrectomy				0.945
Yes	146 (79.3)	30 (79.0)	116 (79.5)	
No	38 (20.7)	8 (21.0)	30 (20.5)	
History of cytokine treatment				0.811
Yes	66 (35.9)	13 (34.2)	53 (36.3)	
No	118 (64.1)	25 (65.8)	93 (63.7)	
Fuhrman grade				<0.001
1–2	104 (56.5)	9 (23.7)	95 (65.1)	
3–4	65 (35.3)	24 (63.2)	41 (28.1)	
Unknown	15 (8.2)	5 (13.1)	10 (6.8)	
Number of metastatic sites				0.053
1	130 (70.7)	22 (57.9)	108 (74.0)	
≥2	54 (29.3)	16 (42.1)	38 (26.0)	
Metastatic site				
Lung	137 (74.5)	33 (86.8)	104 (71.2)	0.050
Lymph node	44 (23.9)	11 (29.0)	33 (22.6)	0.414
Bone	20 (10.9)	0 (0.0)	20 (13.7)	0.034
Liver	16 (8.7)	2 (5.3)	14 (9.6)	0.400
Others	15 (8.2)	2 (5.3)	13 (8.9)	0.465
MSKCC risk category				0.072
Favorable	83 (45.1)	12 (31.6)	71 (48.6)	
Intermediate	72 (39.1)	18 (47.4)	54 (37.0)	
Poor	29 (15.8)	8 (21.0)	21(14.4)	
NLR				0.440
<2.2	73 (39.7)	13 (34.2)	60 (41.1)	
≥2.2	111 (60.3)	25 (65.8)	86 (58.9)	
First-line therapy				0.149
Sorafenib	112 (60.9)	27 (71.1)	85 (58.2)	
Sunitinib	72 (39.1)	11 (28.9)	61 (41.8)	

ccRCC, clear cell renal cell carcinoma; nccRCC, non-clear cell renal cell carcinoma; MSKCC, Memorial Sloan Kettering Cancer Center; NLR, neutrophil-to-lymphocyte ratio

### Association of hypoalbuminemia with clinicopathologic characteristics

**Fig. 1:**
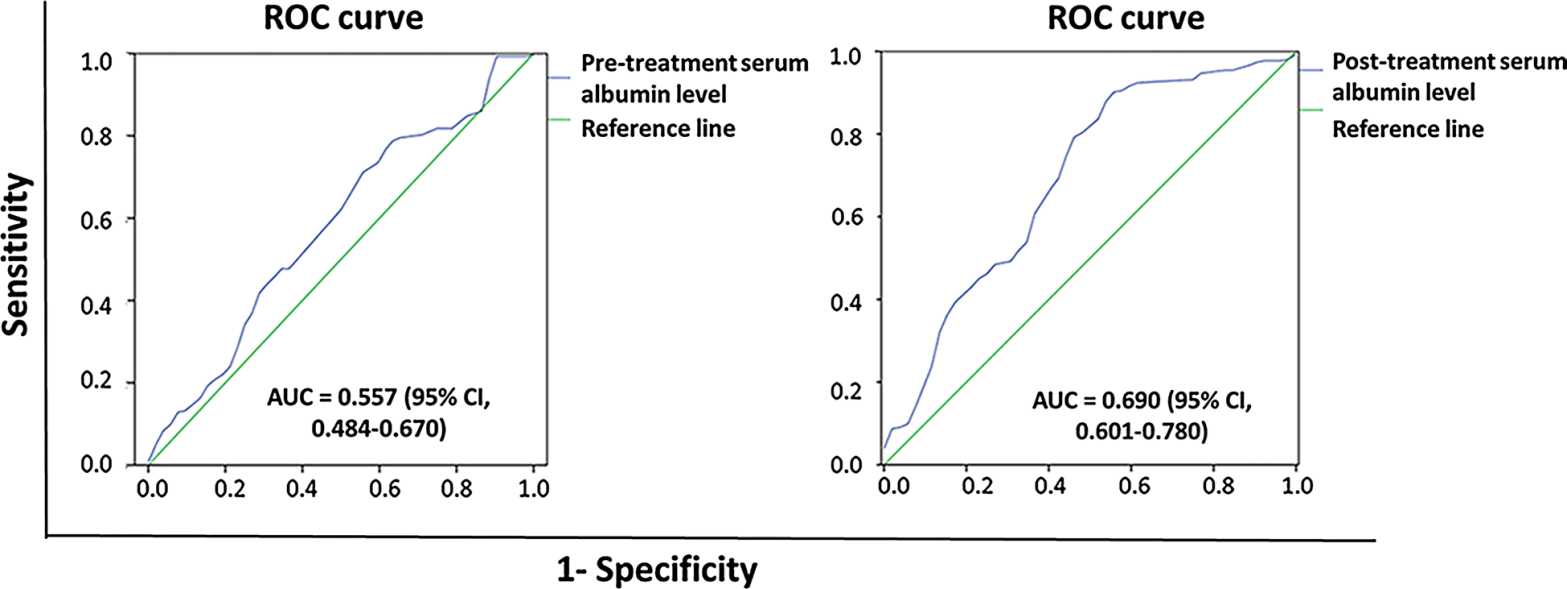
Receiver operating characteristic (ROC) analysis to predict the cutoff levels of pre-treatment and post-treatment serum albumin levels. AUC, area under the curve; CI, confidence interval

The median pre-treatment and post-treatment serum albumin levels were 43.1 g/L (range 15.2–57.7 g/L) and 42.6 g/L (range 13.9–54.4 g/L), respectively. ROC analysis showed that the best cutoffs of pre-treatment and post-treatment serum albumin levels were 40.7 g/L (area under the curve [AUC] = 0.557, 95% confidence interval [CI] 0.484–0.670) and 36.4 g/L (AUC = 0.690, 95% CI 0.601–0.780), respectively (Fig. 1). No significant differences were observed in sex, age, pathologic type, history of nephrectomy or surgery, MSKCC risk category, NLR, or type of first-line targeted agents between the post-treatment hypoalbuminemia group and the normal post-treatment albumin level group. More patients had Fuhrman grade 3–4 RCC in the post-treatment hypoalbuminemia group (63.2%, 24/38) than in the post-treatment normal albumin level group (28.1%, 41/146; *P* < 0.001) (Table 1).

### Associations of hypoalbuminemia and clinicopathologic characteristics with survival

**Fig. 2:**
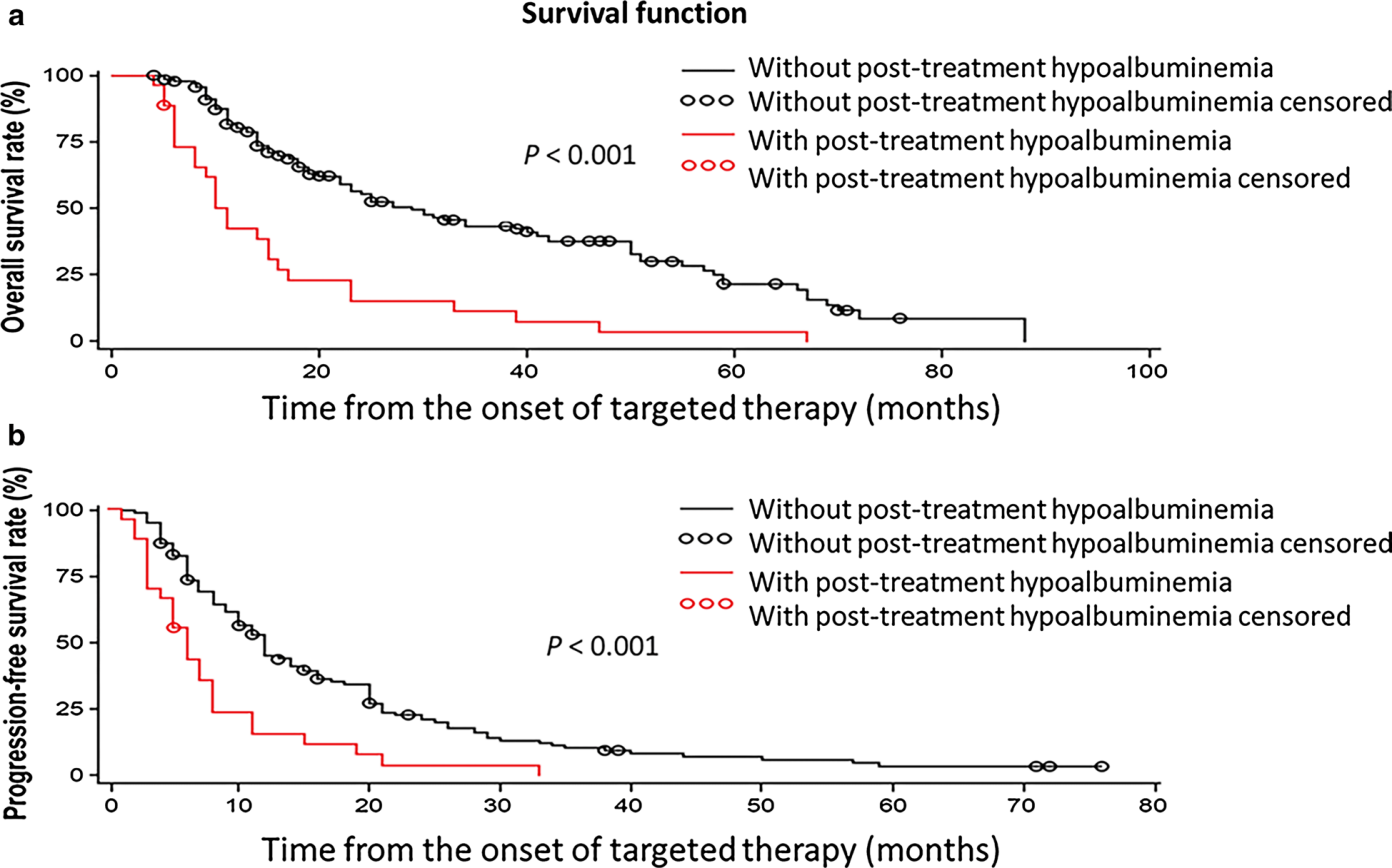
Overall survival (OS) and progression-free survival (PFS) curves of patients with metastatic renal cell carcinoma with and without post-treatment hypoalbuminemia who received sorafenib or sunitinib as first-line therapy. In the post-treatment hypoalbuminemia group, OS (**a**) and PFS (**b**) rates are significantly lower than those in the normal post-treatment albumin level group

Figure 2 shows the Kaplan–Meier curves of PFS and OS. In the post-treatment hypoalbuminemia group, median PFS (6 months; 95% CI 5–7 months) and median OS (11 months; 95% CI 9–15 months) were significantly shorter than those in the normal post-treatment albumin level group (PFS: 12 months, 95% CI 11–16 months, *P* < 0.001; OS: 31 months, 95% CI 24–42 months, *P* < 0.001).

Tables 2 and 3 shows the prognostic value of post-treatment serum albumin level as a continuous variable on PFS and OS when analyzed using the Cox proportional hazards regression model. PFS and OS were not significantly related to sex and age of patients or pathologic type, history of cytokine therapy or nephrectomy, or drug categories. Univariate analysis results showed that Fuhrman grade (*P* = 0.027), number of metastatic sites (*P* = 0.001), MSKCC risk category (*P* < 0.001), NLR (*P* < 0.001), and post-treatment serum albumin level (*P* < 0.001) significantly affected PFS, whereas pre-treatment serum albumin level was not significantly associated with PFS (*P* = 0.075) (Table 2). Additionally, in univariate analysis, OS was significantly associated with Fuhrman grade (*P* < 0.001), number of metastatic sites (*P* < 0.001), MSKCC risk category (*P* < 0.001), NLR (*P* < 0.001), pre-treatment serum albumin level (*P* = 0.012), and post-treatment serum albumin level (*P* < 0.001) (Table 3). Multivariate analysis showed that post-treatment serum albumin level as a continuous variable was a significant risk factor of PFS (hazard ratio [HR] 0.975, 95% CI 0.952–0.999, *P* = 0.042) and OS (HR 0.967, 95% CI 0.941–0.994, *P* = 0.016). Number of metastatic sites, MSKCC risk category, and NLR were also significant risk factors for PFS and OS in multivariate analysis. Fuhrman grade was not significantly associated with PFS but was a prognostic factor for OS in multivariate analysis.

**Table 2:** Prognostic values of clinical variables for predicting progression-free survival in 184 patients with metastatic renal cell carcinoma (including continuous variables) analyzed by using univariate and multivariate Cox regression models

Variable	Univariate		Multivariate	
	HR (95% CI)	*P* value	HR (95% CI)	*P* value
Sex (men vs. women)	1.097 (0.764–1.575)	0.616		
Age (<65 vs. ≥65 years)	1.005 (0.990–1.020)	0.535		
Pathologic type (ccRCC vs. nccRCC)	1.081 (0.690–1.694)	0.733		
History of nephrectomy (yes vs. no)	1.180 (0.790–1.762)	0.418		
History of cytokine treatment (yes vs. no)	1.249 (0.896–1.742)	0.190		
Fuhrman grade (1–2 vs. 3–4 and unknown)	1.298 (1.031–1.633)	0.027*	1.211 (0.951–1.542)	0.121
Number of metastatic sites (1 vs. ≥ 2)	1.763 (1.255–2.476)	0.001*	1.576 (1.092–2.274)	0.015
MSKCC risk category (favorable and intermediate vs. poor)	1.927 (1.548–2.399)	<0.001*	1.957 (1.558–2.460)	0.004
NLR (continuous variable)	1.133 (1.061–1.209)	<0.001*	1.103 (1.032–1.178)	0.004
Drug category (sorafenib vs. sunitinib)	1.020 (0.739–1.408)	0.905		
Pre-treatment serum albumin level (continuous variable)	0.975 (0.949–1.003)	0.075		
Post-treatment serum albumin level (continuous variable)	0.961 (0.941–0.982)	<0.001*	0.975 (0.952–0.999)	0.042

ccRCC, clear cell renal cell carcinoma; nccRCC, non-clear cell renal cell carcinoma; NLR, neutrophil-to-lymphocyte ratio; MSKCC, Memorial Sloan Kettering Cancer Center; HR, hazard ratio; CI, confidence interval

* Variables with *P* values <0.05 in univariate analysis were considered for multivariate analysis

**Table 3:** Prognostic values of clinical variables for predicting overall survival in 184 patients with metastatic renal cell carcinoma (including continuous variables) analyzed by using univariate and multivariate Cox regression models

Variable	Univariate		Multivariate	
	HR (95% CI)	*P* value	HR (95% CI)	*P* value
Sex (men vs. women)	1.204 (0.806–1.799)	0.363		
Age (<65 vs. ≥65 years)	0.999 (0.983–1.016)	0.934		
Pathologic type (ccRCC vs. nccRCC)	1.408 (0.896–2.221)	0.138		
History of nephrectomy (yes vs. no)	1.103 (0.691–1.760)	0.681		
History of cytokine treatment (yes vs. no)	0.963 (0.667–1.391)	0.842		
Fuhrman grade (1–2 vs. 3–4 and unknown)	1.620 (1.260–2.081)	<0.001*	1.491 (1.147–1.937)	0.003
Number of metastatic sites (1 vs. ≥ 2)	2.010 (1.384–2.918)	<0.001*	1.734 (1.162–2.588)	0.007
MSKCC risk category (favorable and intermediate vs. poor)	1.927 (1.548–2.399)	<0.001*	2.236 (1.747–2.862)	<0.001
NLR (continuous variable)	1.163 (1.085–1.247)	<0.001*	1.113 (1.034–1.199)	0.005
Drug category (sorafenib vs. sunitinib)	1.036 (0.725–1.480)	0.847		
Pre-treatment serum albumin level (continuous variable)	0.962 (0.934–0.992)	0.012*	1.008 (0.970–1.047)	0.691
Post-treatment serum albumin level (continuous variable)	0.952 (0.930–0.974)	<0.001	0.967 (0.941–0.994)	0.016

ccRCC, clear cell renal cell carcinoma; nccRCC, non-clear cell renal cell carcinoma; NLR, neutrophil-to-lymphocyte ratio; MSKCC, Memorial Sloan Kettering Cancer Center; HR, hazard ratio; CI, confidence interval

* Variables with *P* values <0.05 in univariate analysis were considered for multivariate analysis

Furthermore, Tables 4 and 5 shows the prognostic value of post-treatment serum albumin level divided by best cutoff point in the Cox proportional hazards regression model. Univariate analysis showed that Fuhrman grade (*P* < 0.001), number of metastatic sites (*P* < 0.001), MSKCC risk category (*P* < 0.001), NLR (*P* = 0.002), pre-treatment serum albumin level (*P* = 0.031), and post-treatment serum albumin level (*P* < 0.001) were significantly associated with PFS (Table 4). Additionally, in univariate analysis, OS was significantly associated with Fuhrman grade (*P* = 0.001), number of metastatic sites (*P* < 0.001), MSKCC risk category (*P* < 0.001), NLR (*P* = 0.002), and post-treatment hypoalbuminemia (*P* < 0.001) (Table 5). Multivariate analysis showed that post-treatment serum albumin level was a significant risk factor of PFS (HR 2.113, 95% CI 1.390–3.212, *P* < 0.001) and OS (HR 2.388, 95% CI 1.591–3.585, *P* < 0.001). Meanwhile, number of metastatic sites, MSKCC risk category, and NLR were also significant risk factors for PFS and OS. However, Fuhrman grade was not significantly associated with PFS but was a prognostic factor of OS in multivariate analysis.

**Table 4:** Prognostic values of clinical variables for predicting progression-free survival in 184 patients with metastatic renal cell carcinoma (all categorical variables) analyzed by using univariate and multivariate Cox regression models

Variable	Univariate		Multivariate	
	HR (95% CI)	*P* value	HR (95% CI)	*P* value
Sex (men vs. women)	1.204 (0.806–1.799)	0.363		
Age (<65 vs. ≥65 years)	0.999 (0.983–1.016)	0.934		
Pathologic type (ccRCC vs. nccRCC)	1.408 (0.896–2.221)	0.138		
History of nephrectomy (yes vs. no)	1.103 (0.691–1.760)	0.681		
History of cytokine treatment (yes vs. no)	0.963 (0.667–1.391)	0.842		
Fuhrman grade (1–2 vs. 3–4 and unknown)	1.620 (1.260–2.081)	<0.001*	1.181 (1.147–1.937)	0.196
Number of metastatic sites (1 vs. ≥ 2)	2.010 (1.384-2.918)	<0.001*	1.601 (1.110–2.311)	0.012
MSKCC risk category (favorable and intermediate vs. poor)	1.927 (1.548–2.399)	<0.001*	2.000 (1.060–2.154)	<0.001
NLR (<2.2 vs. ≥2.2)	1.679 (1.201–2.347)	0.002*	1.511 (1.060–2.154)	0.022
Drug category (sorafenib vs. sunitinib)	1.036 (0.725–1.480)	0.847		
Pre-treatment serum albumin level (≥40.7 g/L vs. <40.7 g/L)	1.432 (1.034–1.984)	0.031*	0.913 (0.631–1.319)	0.627
Post-treatment serum albumin level (≥36.4 g/L vs. <36.4 g/L)	2.392 (1.637–3.494)	<0.001*	2.113 (1.390–3.212)	<0.001

ccRCC, clear cell renal cell carcinoma; nccRCC, non-clear cell renal cell carcinoma; NLR, neutrophil-to-lymphocyte ratio; MSKCC, Memorial Sloan Kettering Cancer Center; HR, hazard ratio; CI, confidence interval

* Variables with *P* values <0.05 in univariate analysis were considered for multivariate analysis

**Table 5:** Prognostic values of clinical variables for predicting overall survival in 184 patients with metastatic renal cell carcinoma (all categorical variables) analyzed by using univariate and multivariate Cox regression models

Variable	Univariate		Multivariate	
	HR (95% CI)	*P* value	HR (95% CI)	*P* value
Sex (men vs. women)	1.204 (0.806–1.799)	0.363		
Age (<65 vs. ≥65 years)	0.999 (0.983–1.016)	0.934		
Pathologic type (ccRCC vs. nccRCC)	1.408 (0.896–2.221)	0.138		
History of nephrectomy (yes vs. no)	1.103 (0.691–1.760)	0.681		
History of cytokine treatment (yes vs. no)	0.963 (0.667–1.391)	0.842		
Fuhrman grade (1–2 vs. 3–4 and unknown)	1.620 (1.260–2.081)	<0.001*	1.495 (1.134–1.974)	0.004
Number of metastatic sites (1 vs. ≥ 2)	2.010 (1.384–2.918)	<0.001*	1.736 (1.163–2.592)	0.007
MSKCC risk category (favorable and intermediate vs. poor)	1.927 (1.548–2.399)	<0.001*	2.248 (1.759–2.871)	<0.001
NLR (<2.2 vs. ≥2.2)	1.679 (1.201–2.347)	0.002*	1.804 (1.184–2.750)	0.006
Drug category (sorafenib vs. sunitinib)	1.036 (0.725–1.480)	0.847		
Pre-treatment serum albumin level (≥40.7 g/L vs. <40.7 g/L)	1.407 (0.977–2.027)	0.066		
Post-treatment serum albumin level (≥36.4 g/L vs. <36.4 g/L)	2.392 (1.637–3.494)	<0.001*	2.388 (1.591–3.585)	<0.001

ccRCC, clear cell renal cell carcinoma; nccRCC, non-clear cell renal cell carcinoma; NLR, neutrophil-to-lymphocyte ratio; MSKCC, Memorial Sloan Kettering Cancer Center; HR, hazard ratio; CI, confidence interval

* Variables with *P* values <0.05 in univariate analysis were considered for multivariate analysis

Table 6 shows the predictive accuracy of the basic MSKCC risk model and with the integrated MSKCC risk model involving post-treatment hypoalbuminemia. The predictive accuracy of the basic MSKCC risk model was 0.67 (95% CI 0.62–0.72) for PFS and 0.70 (95% CI 0.65–0.75) for OS; after adding hypoalbuminemia (36.4 g/L) to the basic MSKCC risk model, the predictive accuracy was improved to 0.68 (95% CI 0.63–0.73) for PFS and 0.73 (95% CI 0.67–0.79) for OS. In a model including all significant variables in the present study (Fuhrman grade, number of metastatic sites, MSKCC risk category, NLR, and post-treatment hypoalbuminemia), the predictive accuracy was further improved to 0.72 (95% CI 0.66–0.78) for PFS and 0.79 (95% CI 0.73–0.85) for OS (Table 6).

**Table 6:** Comparison of the survival predictive power of basic MSKCC risk model and integrated model involving post-treatment hypoalbuminemia

Variable	C-index^a^	
	PFS	OS
Basic MSKCC risk model	0.67	0.70
Integrated MSKCC risk model involving NLR	0.69	0.72
Integrated MSKCC risk model involving post-treatment hypoalbuminemia	0.68	0.73
Integrated MSKCC risk model involving all significant variables^b^	0.72	0.79

PFS, progression-free survival; OS, overall survival; C-index, Harrell concordance index

^a^The standard deviations for all these values are 0.03

^b^All significant variables include Fuhrman grade, number of metastatic sites, MSKCC risk category, NLR, and post-treatment hypoalbuminemia

We performed ROC analysis and found that the integrated MSKCC risk model involving post-treatment hypoalbuminemia (AUC = 0.678, 95% CI 0.601–0.754; AUC = 0.759 95% CI 0.674–0.844) showed better predictive value than the basic MSKCC risk model (AUC = 0.605, 95% CI 0.521–0.686; AUC = 0.658, 95% CI 0.563–0.753) for PFS (*P* < 0.001) and OS (*P* < 0.001), respectively.

**Fig. 3:**
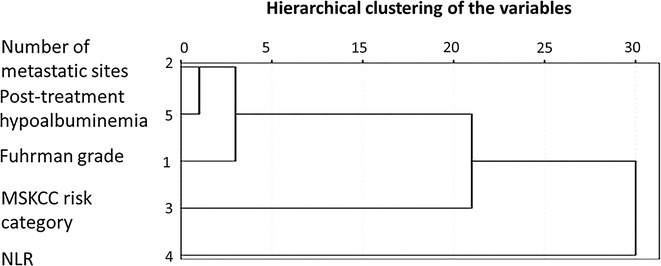
Hierarchical clustering of the variables associated with post-treatment hypoalbuminemia. The hierarchy dendrogram of five significant prognostic factors using average linkage method indicates that post-treatment hypoalbuminemia is most highly associated with number of metastatic sites. NLR, neutrophil-to-lymphocyte ratio; MSKCC, Memorial Sloan Kettering Cancer Center

The hierarchy dendrogram of five significant prognostic factors mentioned above using average linkage method indicated that post-treatment hypoalbuminemia was most highly associated with number of metastatic sites. Furthermore, Fuhrman grade, MSKCC risk category, and NLR could be combined to predict prognosis (Fig. 3).

### Safety assessments

During the study, no serious adverse events were observed in the two groups (Table 7). Common adverse events after sorafenib or sunitinib initiation were hand–foot syndrome (65.8%), diarrhea (53.3%), fatigue (42.4%), nausea (40.2%), and hypertension (32.1%). The adverse events were usually mild to moderate (grade 1 or 2; range 8.7%–60.6%); however, some adverse events were severe (grade 3–4; 0.0%–4.9%). Commonly observed grade 3–4 adverse events after sorafenib or sunitinib initiation were hand-foot syndrome (4.9%), diarrhea (2.7%), and hypertension (2.2%).

**Table 7:** Summary of drug-related adverse events in the 184 patients with metastatic renal cell carcinoma

Drug-related adverse event	None	Any grade	Grade 1–2	Grade 3–4
Hand-foot syndrome	63 (34.2)	121(65.8)	112 (60.6)	9 (4.9)
Diarrhea	86 (46.7)	98 (53.3)	93 (50.5)	5 (2.7)
Nausea	110 (59.8)	74 (40.2)	74 (40.2)	0 (0.0)
Fatigue	106 (57.6)	78 (42.4)	78 (42.4)	0 (0.0)
Hypertension	125 (69.6)	59 (32.1)	55 (29.9)	4 (2.2)
Thrombocytopenia	144 (78.3)	40 (21.7)	38 (20.7)	2 (1.1)
Leukocytopenia	147 (79.9)	37 (20.1)	35 (19.0)	2 (1.1)
Anemia	156 (84.8)	28 (15.2)	26 (14.1)	2 (1.1)
Elevation of ALT	160 (87.0)	24 (13.0)	23 (11.5)	1 (0.5)
Alopecia	167 (90.8)	17 (9.2)	17 (9.2)	0 (0.0)
Hypothyroidism	168 (91.3)	16 (8.7)	16 (8.7)	0 (0.0)

All data are presented as the number of patients followed by percentage in parentheses

ALT, alanine aminotransferase

Anemia was significantly more common in the post-treatment hypoalbuminemia group than in the post-treatment normal serum albumin level group (29.6% vs. 11.0%, *P* = 0.002), whereas the proportions of hand-foot syndrome, diarrhea, nausea, fatigue, hypertension, thrombocytopenia, leukocytopenia, alopecia, elevation of alanine aminotransferase, and hypothyroidism showed no significant differences between two groups (Table 8).

**Table 8:** Drug-related adverse events in metastatic renal cell carcinoma patients with post-treatment hypoalbuminemia and normal serum albumin level

Drug-related adverse event	Post-treatment hypoalbuminemia (albumin level < 36.4 g/L) group			Normal serum albumin level (albumin level ≥36.4 g/L) group			*P* value*
	Any grade	Grade 1–2	Grade 3–4	Any grade	Grade 1–2	Grade 3–4	
Hand–foot syndrome	22 (57.9)	22 (57.9)	0 (0.0)	99 (67.8)	90 (61.6)	9 (6.2)	0.251
Diarrhea	20 (52.6)	20 (52.6)	0 (0.0)	78 (53.4)	73 (50.0)	5 (3.4)	0.930
Nausea	20 (52.6)	20 (52.6)	0 (0.0)	54 (37.0)	54 (37.0)	0 (0.0)	0.080
Fatigue	17 (44.7)	17 (44.7)	0 (0.0)	61 (41.8)	61 (41.8)	0 (0.0)	0.743
Hypertension	17 (44.7)	17 (44.7)	0 (0.0)	42 (28.7)	38 (26.0)	4 (2.7)	0.060
Anemia	12 (29.6)	10 (26.3)	2 (5.3)	16 (11.0)	16 (11.0)	0 (0.0)	0.002
Thrombocytopenia	11 (29.0)	11 (29.0)	0 (0.0)	29 (19.9)	27 (18.5)	2 (1.4)	0.227
Leukocytopenia	8 (21.1)	8 (21.1)	0 (0.0)	29 (19.9)	27 (18.5)	2 (1.4)	0.871
Elevation of ALT	8 (21.0)	8 (21.1)	0 (0.0)	16 (11.0)	15 (10.3)	1 (0.7)	0.170
Alopecia	6 (15.8)	6 (15.8)	0 (0.0)	11 (7.5)	11 (7.5)	0 (0.0)	0.211
Hypothyroidism	3 (7.9)	3 (7.9)	0 (0.0)	13 (8.9)	13 (8.9)	0 (0.0)	1.000

All data are presented as the number of patients followed by percentage in parentheses

ALT, alanine aminotransferase

* Grade 1–2 and grade 3–4 adverse events were combined in *P* value calculation

## Discussion

In this study, we investigated the association between post-treatment albumin level and survival of mRCC patients who received first-line targeted therapy with TKIs (sorafenib or sunitinib). We found that post-treatment hypoalbuminemia was independently associated with shorter PFS and OS in these mRCC patients. Additionally, we found that number of metastatic sites, MSKCC risk category, and NLR were independent predictors of OS and PFS; Fuhrman grade was a prognostic factor for OS but not for PFS.

In a previous study, malnutritional status was shown to be a high risk factor in patients with localized RCC [18]. Recently, Gu et al. [19] reported that mRCC patients who received targeted therapy and who had low nutritional assessment scores had a poor prognosis. In their study, they classified risk of malnutrition according to the Geriatric Nutritional Risk Index (GNRI) and the Mini Nutritional Assessment-Short Form (MNA-SF): MNA-SF scores for assessing nutritional deficiency are partly based on patients’ memory, whereas GNRI is an objective parameter as it is calculated using the formula $1. 4 8 9\; \times \;{\text{albumin }}\left( {{\text{g}}/{\text{L}}} \right) + 4 1. 7\; \times \; \left( {{\text{weight}}/{\text{ideal body weight}}} \right)$ [19]. One indicator of GNRI is serum albumin level, which is a simple criterion to indicate a patient’s nutritional status [31]. Low serum albumin levels could predict poor survival outcomes in patients with RCC [32, 33]. For RCC patients, low levels of preoperative serum albumin were associated with a high rate of blood transfusion during radical nephrectomy [25]. Moreover, hypoalbuminemia shortened the OS and PFS of patients with mRCC who received cytoreductive nephrectomy [26]. Stenman et al. [27] reported that pre-treatment serum albumin level was independently associated with outcomes in mRCC patients who received TKI treatment (HR = 2.72, *P* = 0.015). In a systematic review, Gupta et al. [31] reported that pre-treatment hypoalbuminemia was associated with shortened survival of patients with RCC. Yildiz et al. [28] found that pre-treatment hypoalbuminemia predicted short PFS and OS in Turkish patients with mRCC who received once-daily continuous administration of first-line sunitinib. However, in the present study, we found that post-treatment hypoalbuminemia, but not low pre-treatment serum albumin level, was a risk factor that predicted poor outcome for mRCC patients who received sorafenib or sunitinib. The reasons may be as follows: first, patients had eating difficulty because of TKI-related adverse effects such as mouth ulcers, which might have led to their poor nutritional status; second, some patients might also have experienced deterioration in nutritional status after treatment. Therefore, post-treatment hypoalbuminemia may be a better prognostic factor which can reflect the nutritional status after targeted therapy than pre-treatment serum albumin level in patients with mRCC. In the present study, we found that pretreatment serum albumin level was significant for PFS in univariate analysis (*P* = 0.031) but not in multivariate analysis (*P* = 0.627).

In addition to the role in patient nutritional status, peripheral serum albumin level has also been reported to be significantly associated with the host immune system and tumor progression. Fox et al. [34] suggested that serum albumin level is an inflammatory marker, adding significance to the basic MSKCC risk model. Recently, NLR was shown to be an important inflammation-related prognostic factor for mRCC patients who receive targeted therapy [14]. In the present study, although no significant difference in NLR was observed between the post-treatment hypoalbuminemia group and the normal post-treatment serum albumin group, a higher NLR was observed in the post-treatment hypoalbuminemia group than in the normal post-treatment albumin level group, suggesting that, to some degree, a low post-treatment serum albumin level is associated with systemic inflammation. Future studies need to explore the influence of these two factors on each other.

This study had several limitations, and the data should be interpreted cautiously. First, this was a retrospective study from a single center with a relatively small sample size; this indicates the possibility of confounding data and probable bias, leading to skewed results of the analysis. Second, additional nutrition-related prognostic factors, such as weight loss and lymphocyte count, were not considered. Moreover, some patients switched to other targeted drugs if they experienced disease progression. Third, post-treatment hypoalbuminemia may deteriorate over time; thus, long-term studies are warranted to evaluate the prognostic value of this time-dependent variable. Also, patients with low BMI will have a low serum albumin level after treatment with TKIs; therefore, additional studies that balance BMI and serum albumin levels of patients should be conducted. Future translational studies, including large-scale, long-term randomized studies, are warranted to validate the findings of our study. In addition, to develop novel prognostic criteria for mRCC, researchers may study the underlying mechanisms by which post-treatment serum albumin level influences the efficacy and tolerability of targeted therapy for mRCC patients as well as the dynamic variation of serum albumin levels after they receive targeted therapy.

## Conclusions

We found that post-treatment hypoalbuminemia was a significant prognostic factor to predict short PFS and OS in patients with mRCC who received sunitinib or sorafenib as first-line targeted therapy; integrating post-treatment serum albumin level into the basic MSKCC risk model may improve the accuracy of the MSKCC model in predicting patient overall survival and progression-free survival. Our findings suggest that post-treatment hypoalbuminemia could be an underlying target for improving survival of patients with mRCC and reflect the current treatment paradigm of mRCC. This may also help determine treatment modalities to improve patient outcomes and better stratify patients in clinical trials.
